# GWAS Uncovers Differential Genetic Bases for Drought and Salt Tolerances in Sesame at the Germination Stage

**DOI:** 10.3390/genes9020087

**Published:** 2018-02-14

**Authors:** Donghua Li, Komivi Dossa, Yanxin Zhang, Xin Wei, Linhai Wang, Yujuan Zhang, Aili Liu, Rong Zhou, Xiurong Zhang

**Affiliations:** 1 Oil Crops Research Institute of the Chinese Academy of Agricultural Sciences, Key Laboratory of Biology and Genetic Improvement of Oil Crops, Ministry of Agriculture, No.2 Xudong 2nd Road, Wuhan 430062, China; ldh3606810@163.com (D.L.); dossakomivi@gmail.com (K.D.); zhangyanxin@caas.cn (Y.Z.); weixin@caas.cn (X.W.); linhai827@163.com (L.W.); zhangyujuan@caas.cn (Y.Z.); liuailihappy@126.com (A.L.); rongzzzzzz@126.com (R.Z.); 2 Centre d’Etudes Régional Pour l’Amélioration de l’Adaptation à la Sécheresse (CERAAS), Route de Khombole, Thiès, BP 3320, Senegal; dossakomivi@gmail.com; 3 College of Life and Environmental Sciences, Shanghai Normal University, Shanghai 200234, China

**Keywords:** *Sesamum indicum*, drought stress, salt stress, GWAS, genetic variants, candidate genes

## Abstract

Sesame has great potential as an industrial crop but its production is challenged by drought and salt stresses. To unravel the genetic variants leading to salinity and drought tolerances at the germination stage, genome-wide association studies of stress tolerance indexes related to NaCl-salt and polyethylene glycol-drought induced stresses were performed with a diversity panel of 490 sesame accessions. An extensive variation was observed for drought and salt responses in the population and most of the accessions were moderately tolerant to both stresses. A total of 132 and 120 significant Single Nucleotide Polymorphisms (SNPs) resolved to nine and 15 Quantitative trait loci (QTLs) were detected for drought and salt stresses, respectively. Only two common QTLs for drought and salt responses were found located on linkage groups 5 and 7, respectively. This indicates that the genetic bases for drought and salt responses in sesame are different. A total of 13 and 27 potential candidate genes were uncovered for drought and salt tolerance indexes, respectively, encoding transcription factors, antioxidative enzymes, osmoprotectants and involved in hormonal biosynthesis, signal transduction or ion sequestration. The identified SNPs and potential candidate genes represent valuable resources for future functional characterization towards the enhancement of sesame cultivars for drought and salt tolerances.

## 1. Introduction

Sesame (*Sesamum indicum* L.) is a traditional crop considered as one of the oldest oilseed crops known by mankind [1]. It plays a very significant role in preserving food and nutritional security as well as livelihood improvement in developing regions of the world. Over the last decade, the production of sesame seeds has doubled, showing the increasing interest on this crop [2]. Sesame is especially significant in the arid and semi-arid regions where the cultivation of several major crops including maize, cotton, etc., becomes problematic. In fact, salinity and water deficit have a strong influence on agricultural development in these regions [3]. Because of the low rainfall coupled with an intense heat, drought frequently occurs and greatly impairs crop productivity. On the other hand, intense use of irrigation and applied fertilizers are the major causes of soil salinity which inhibits seed germination and plant growth, resulting in a drastic reduction of yields [4]. In contrast to most of the oilseed crops, sesame is a fairly resilient crop reported to be relatively tolerant to drought [5] and also rated moderately salt tolerant [6]. These features raise sesame as an attractive crop tailored for the challenging environmental conditions of the arid and semi-arid regions.

Despite sesame’s relative drought tolerance, severe water deficit constitutes a major impediment for its production. Especially, drought stress occurring at germination and anthesis stages has the most damaging effects on seed germination, seedling development, yield related traits and seed quality [7,8,9,10,11,12]. Similarly, detrimental effects of severe salt stress especially NaCl, on sesame seed germination, seedling growth and yield were documented by several authors [6,13,14,15,16]. Exposures to drought, salt as well as other abiotic and biotic factors are considerably decreasing sesame yield which is actually around 300–400 kg/ha [5] far from its full potential of 2500 kg/ha [17]. Therefore, enhancing sesame genotypes for drought and salinity tolerances constitutes one of the priorities in the current sesame research [2].

Several studies were performed on the screening for sources of tolerance in sesame germplasm in regard to drought and salt stresses [7,9,14,15,16,18,19,20,21,22,23,24,25,26]. It is reported that sesame accessions harbor different degrees of tolerance to these abiotic stresses by triggering various response mechanisms including the control of ion transfer from roots to leaves, the accumulation of osmotic regulators, a strong induction of antioxidative enzymes, the regulation of photosynthesis and phytohormones, etc. [18,19,20,21,22,23,24,25,26]. The diversity in sesame responses to salt and drought stresses are controlled by genetic factors. Therefore, understanding the genetic components for salt and drought tolerances by identifying the genetic loci and the candidate genes associated with these traits would be an economical, feasible and efficient way to accelerate the progress of abiotic tolerance breeding in sesame [2]. Seed germination is one the most sensitive stages of sesame growth cycle [9]. Accordingly, detecting some genetic variants associated with sesame tolerance to drought and salinity at the germination stage will be a major asset for sesame cultivation in arid and semi-arid regions.

Genome Wide Association Study (GWAS) approach is widely employed to identify relationships between molecular markers or candidate genes and traits in a given population based on linkage disequilibrium. It offers advantages over traditional linkage analyses, such as more accurate positioning and mapping, simultaneous assessments of multiple alleles at a locus and no requirement for linkage group construction [27,28]. It has been successfully applied in several crops such as alfalfa, *Arabidopsis thaliana*, soybean, maize, rice, asparagus bean, *Brassica napus*, etc., to dissect complex traits including salt and drought tolerance [29,30,31,32,33,34,35,36,37,38,39]. GWAS was also successfully applied in sesame to unravel the genetic basis of its high oil production and quality, and some other key agronomic traits by fully sequencing a population composed of 705 worldwide accessions [40]. The release of the genotypic data of this panel provided inestimable genomic information to conduct further GWAS on other important traits in sesame. This study aimed at identifying the genetic bases of salt (NaCl-induced) and drought (polyethylene glycol (PEG)-induced) tolerances at the seed germination stage in 490 diverse sesame accessions using GWAS.

## 2. Materials and Methods

### 2.1. Plant Materials

Seeds of 490 sesame accessions were obtained from the China National Gene Bank, Oil Crops Research Institute, Chinese Academy of Agricultural Sciences (Wuhan, China). The 490 accessions used for the association analysis in this study were from 33 countries of Asia, Africa, America, and Europe (Table S1), aiming to capture a high geographical diversity. All accessions were self-pollinated for four generations by growing under natural growing conditions in Sanya, Hainan province, China (109.187° E, 18.38° N, altitude 11 m). Seed lots from 2013 harvest were mainly used in this study. However, for the following accessions G656, G041, G051, G123, G128, G162, G175, G176, G189, G204, G208, G213, G215, G224, G228, G233, G242, G262, G264, G299, G301, G315, G352, G391, G400, G401, G407, G413 with limited seed quantities, seed lots from 2013 were used for controls while seed lots from 2014 were used for stress treatments.

### 2.2. Salt and Polyethylene Glycol Stress Treatment

A pilot experiment was conducted in order to identify the suitable concentrations of NaCl and PEG 6000 which could induce most stress conditions and could easily distinguish sesame germplasm according to their tolerance levels. For the NaCl, different concentrations were tested including 0 mM, 40 mM, 60 mM, 80 mM and 100 mM on 15 diverse accessions (Table S2). For each accession, 50 seeds were germinated in petri dishes (90 mm diameter) containing two layers of filter paper (Whatman™, Malaga, WA, Australia). Two treatments were applied: a control condition in which seeds were soaked with 10 mL deionized water; a stress condition in which seeds were soaked with 10 mL of the different concentrations of NaCl. Petri dishes were maintained in the dark into a climatic chamber (SANYO, Gallenkamp PLC, Loughborough, UK) set at 28 °C for five days. Every day, deionized water and the NaCl solutions were added into the petri dishes to compensate for evaporation in the control and stress treatments, respectively. The experiment was arranged in a completely randomized design with three replications for each accession in both treatments. In regard to the PEG 6000, similar procedures were followed as for NaCl. Different concentrations of 0%, 5%, 10%, 15%, 20%, 25% and 30% PEG 6000 were tested on 15 accessions. Then, the numbers of germinated seeds were counted and the germination percentage was estimated through the formula: (number of germinated seeds/number of plated seeds (50)) × 100.

For the 15 accessions tested, a mean germination percentage was estimated. According to the suitable concentrations identified for NaCl and PEG 6000 treatments in the pilot experiment, the 490 accessions were submitted to 60 mM NaCl and 15% PEG 6000 following experimental procedures described above. The whole experiment was repeated three times. After five days, the number of germinated seeds (GR) and the fresh weight (FW (g)) of the seedlings were recorded in both control and stress conditions.

### 2.3. Statistical Analysis

Based on the recorded data, the stress tolerance (ST) index was estimated as the ratio of the number of germinated seeds and fresh weight under stress conditions to the same traits under stress free conditions [41]. Therefore, ST-SGR (%) (Stress tolerance for germination percentage under salt) and ST-SFW (%) (Stress tolerance for fresh weight under salt) represent the salt tolerance index. While, ST-DGR (%) (Stress tolerance for germination percentage under PEG 6000) and ST-DFW (%) (Stress tolerance for fresh weight under PEG 6000) represent drought tolerance index.

For each trait, data were analyzed using analysis of variance (ANOVA) based on General Linear Model procedure in R3.2.0 software with the packages “Ade4” [42] and “Agricolae” [43] considering all effects as fixed. ANOVA results were considered significant at *p* < 0.05 and the mean comparisons were performed using the Tukey honest significant difference (HSD) test. Additionally, the least square means, standard deviation, variance, and descriptive statistics such as the coefficient of variation, range, skewness and kurtosis were estimated. Correlation coefficients among the stress indexes of PEG and NaCl treatments were calculated by Pearson’s method at a significance level of *p* < 0.05 using the “Corrplot” package [44]. The variation of the different traits in the control and stress conditions was represented employing the “ggplot2” package [45]. Pearson dissimilarity coefficients of stress tolerance indexes were used for hierarchical cluster analysis and the dendrogram based on Ward’s method was constructed in Minitab software (Pennsylvania State University, PA, USA).

### 2.4. Phenotype-Genotype Association Analysis

The association panel used in this study along with other 215 sesame accessions was previously fully re-sequenced in works of Wei et al. [40]. A total of 1,005,413 common Single Nucleotide Polymorphisms (SNPs) covering the whole genome with minor allele frequency >0.03 were used for genome wide association analysis in this study. Average Linkage Disequilibrium (LD) region was estimated to 88 kb in the whole genome of sesame [40]. The relative kinship analysis was implemented using the package SPAGeDi (Université Libre de Bruxelles, Brussels, Belgium) [46]. To get insight into the population structure of the association panel, a dendrogram based on simple matching coefficient was constructed using the software package MEGA7 (Temple University, Philadelphia, PA, USA) [47]. In addition, the program STRUCTURE 2.3.4 (Stanford University, Stanford, CA, USA) [48] was used for a Bayesian clustering analysis with the admixture model and correlated allele frequencies. Five runs were performed for each subpopulation K (1 to 10) [49]. The burn-in time and iterations for each run were set to 30,000 and 70,000, respectively and the true K was determined according to the method described by Evanno et al. [50]. Association analysis was performed for ST-SGR, ST-SFW, ST-DGR and ST-DFW using the EMMAX model [51] based on the Mixed Model. The matrix of pair-wise genetic distance derived from simple matching coefficients was used as the variance–covariance matrix of the random effect. Significance was defined at a uniform threshold of *p* < 5.54 × 10^−7^ (−log_10_ (*p*) > 6). The value *r*^2^ derived from linear regressions were calculated to examine the phenotypic variance explained (PVE) of each peak SNPs using the Minitab software (Pennsylvania State University, PA, USA). Before fitting the model, each marker was coded with the value 0 used for the reference allele and the value 1 for the alternative allele.

### 2.5. Mining of Potential Candidate Genes 

A region of 88 kb (LD [40]) including at least three significant SNPs was considered a Quantitative trait locus (QTL). To identify the potential candidate genes around the significant peak SNPs associated with each trait, the full gene list in the QTL was searched using the sesame reference genome information [52]. The corresponding putative homologs of all genes in *A. thaliana* were retrieved from the database SesameFG [53] with cut off *E*-value ≤1 × 10^−40^. We retained the potential candidate genes based on two criteria: (a). the gene is annotated; (b). the gene annotation is related to abiotic stress response and/or the homolog in *Arabidopsis* is characterized as an abiotic stress responsive gene. Gene Ontology (GO) annotation of the potential candidate genes was carried out using Blast2GO tool v.3.1.3 (Biobam, Valencia, Spain) [54] and plotted with WEGO tool (BGI, Shenzhen, China) [55].

## 3. Results

### 3.1. Identification of Suitable Concentrations for NaCl and Polyethylene Glycol Stress Induction

**Figure 1:**
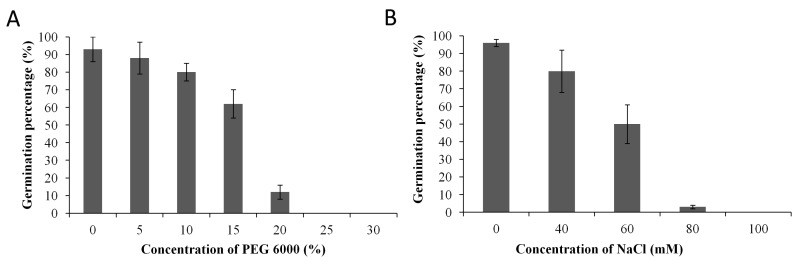
Pilot experiment showing the mean germination percentage of 15 sesame accessions under various concentrations of NaCl and PEG 6000. (**A**) PEG 6000; (**B**) NaCl. PEG: polyethylene glycol.

From 30% to 25% PEG 6000 concentration applications, no seed could germinate, while at 20% concentration, only few seeds (12%) germinated. Therefore, 15% PEG 6000 was defined as the appropriate concentration for examining drought tolerance in the whole germplasm (Figure 1A). Concerning the NaCl concentrations, results showed that 100 mM NaCl was detrimental to the seeds. At 80 mM, only few seeds (3%) germinated; therefore, 60 mM NaCl was selected as the suitable concentration for salt stress tolerance screening in the association panel (Figure 1B). The Figures S1 and S2 present the phenotypes of seedlings from tolerant and sensitive sesame genotypes in responses to PEG 6000 (0% vs. 15%) and NaCl (0 vs. 60 mM), respectively. The growth of the tolerant genotypes was less affected under stress as compared to the sensitive genotypes.

### 3.2. Phenotypic Variation for Salt and Drought Tolerances in the Sesame Germplasm

**Figure 2:**
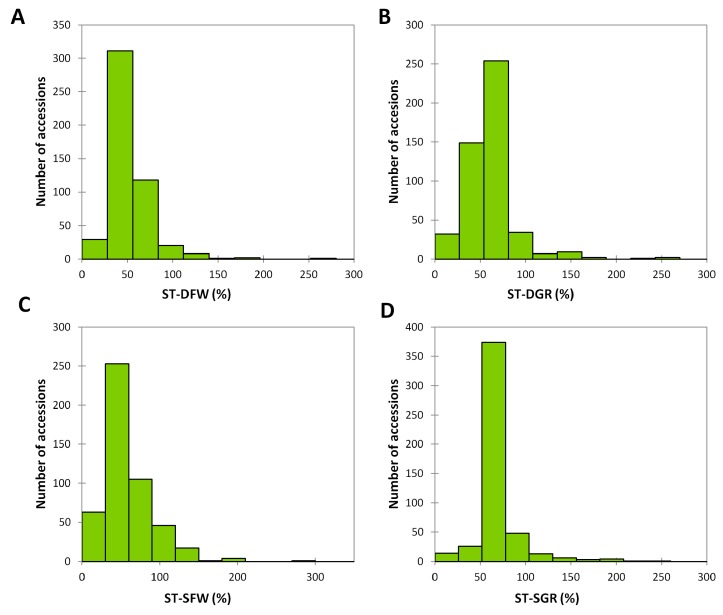
Phenotypic variation of the stress tolerance indexes for the measured traits. (**A**) Stress tolerance for fresh weight under PEG 6000 (ST-DFW) (%); (**B**) Stress tolerance for germination percentage under PEG 6000 (ST-DGR) (%); (**C**) Stress tolerance for fresh weight under salt (ST-SFW) (%); (**D**) Stress tolerance for germination percentage under salt (ST-SGR) (%).

An extensive phenotypic variation was displayed for drought and salt tolerance indexes in the sesame association panel (Figure 2). Normal or nearly normal distributions were observed for all the stress indexes in the mapping population. ST-DFW ranged from 7% to 276% with most of the accessions displaying 50% tolerance. Similarly, ST-DGR ranged from 2% to 266% and most accessions showed 60% tolerance. Concerning the salt stress, a similar trend was observed. ST-SFW values were between 2% and 294% with the majority of the accessions exhibiting 60% tolerance. For the ST-SGR values, most of the accessions displayed a good tolerance (mean ST-SGR value around 70%). Overall, the fresh weight of the seedlings was found to be more affected than the number of germinated seeds, although both traits were significantly (*p* < 0.001) reduced under drought and salt stresses (Table 1). It is worth to mention that for some accessions, the fact that different seed lots were used for controls and stressed conditions, may explain the outperformance of stressed samples over the controls.

**Figure 3:**
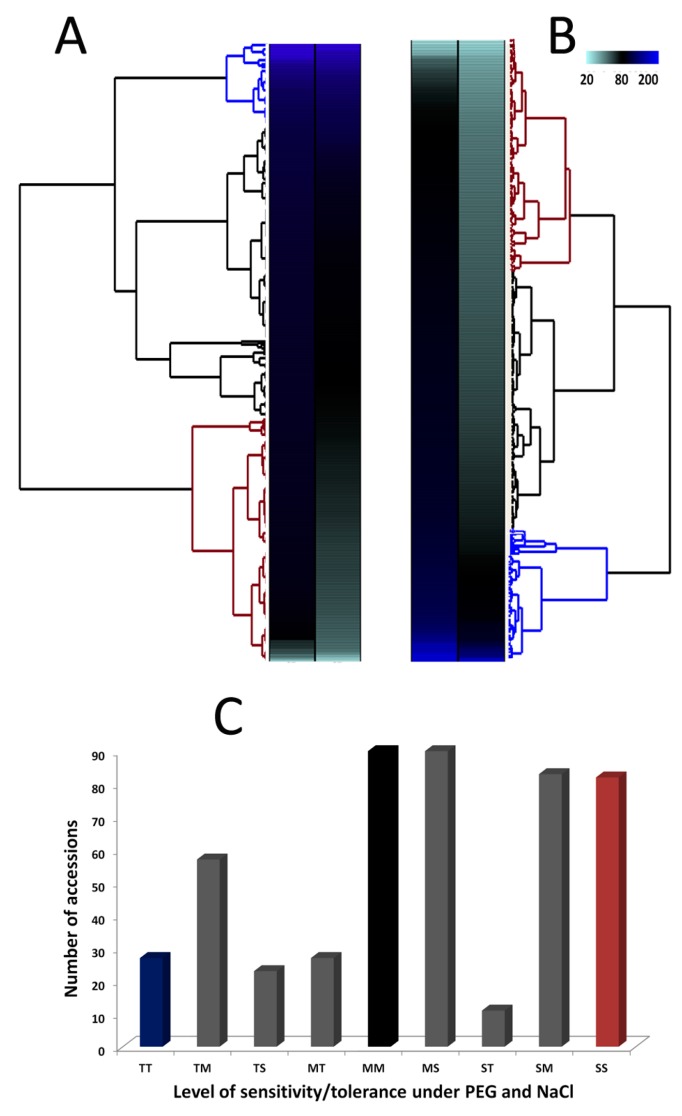
Classification of 490 sesame accessions according to their levels of tolerance/sensitivity to PEG and NaCl stresses. (**A**) Dendrogram and heat map hierarchical clustering of the accessions according to their tolerance levels to NaCl. (**B**) Dendrogram and heat map hierarchical clustering of the accessions according to their tolerance levels to PEG. The cluster colored in carmine gathers the sensitive accessions, the blue one is for the tolerant accessions and the black cluster represents the moderately tolerant accessions. (**C**) Comparison of the responses of the accessions to PEG and NaCl. The bars showed the proportion of accessions according to their responses to both stresses. The bars colored in blue, black and carmine represent the accessions which were commonly tolerant, moderately tolerant and sensitive to both stresses, respectively. The bars colored in gray represent the genotypes displaying contrasting responses to PEG and NaCl treatments. T = Tolerant, M = Moderately tolerant, S = Sensitive, based on the classification of the dendrogram. The first letter refers to PEG while the second letter refers to NaCl.

**Figure 4:**
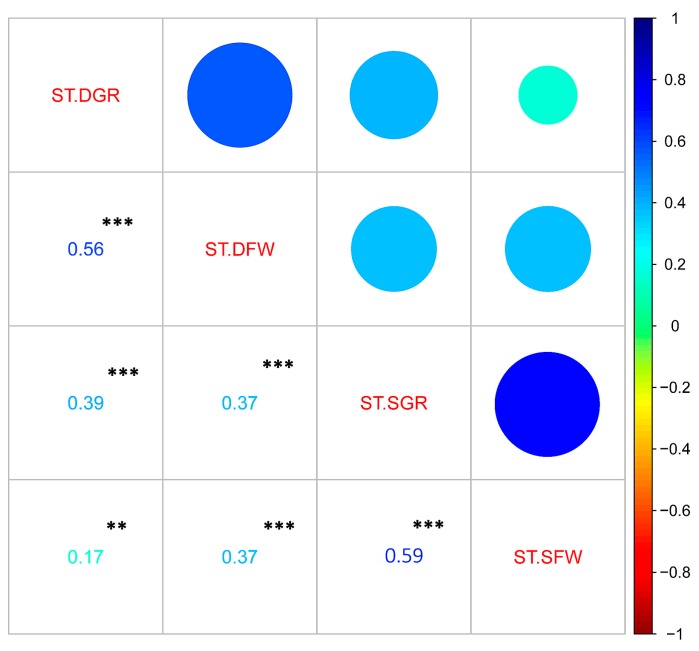
Correlation analysis of the stress tolerance indexes. ***, ** Significant difference at *p* < 0.001 and *p* < 0.01, respectively.

Figure 3A,B presents the clustering patterns of the 490 accessions according to their tolerance levels to salt and drought. In total, three main clusters were observed for both stresses: the cluster of tolerant accessions is colored in blue, the one of moderately tolerant accessions is colored in black and finally the cluster of sensitive accessions is marked in carmine color. In general, the tolerant accessions and sensitive ones were less represented than the moderately tolerant accessions for drought and salt stresses (Table 2). In addition, the responses of sesame accessions to PEG and NaCl are mostly inconsistent (Figure 3C). For instance, only 27 accessions were commonly tolerant to drought and salt stresses (TT), 82 commonly sensitive to both stresses (SS) and 90 accessions identified as commonly moderately tolerant to both stresses (MM). The remaining 291 accessions displayed differential responses to drought and salt stresses (Figure 3C). Similarly, the correlation analysis of the drought and salt tolerance indexes indicated that traits from the same stress were strongly and significantly correlated whereas the correlation of traits between drought and salt were significantly weak (*p* < 0.001) (Figure 4). This indicates that PEG-induced drought stress and NaCl-induced salt stress lead to differential responses in sesame and are probably underlined by different genetic factors.

### 3.3. Genome-Wide Association Studies for Drought and Salt Tolerance Indexes

A total of 1M SNPs were employed for the GWAS analysis of drought and salt tolerance traits, resulting in a high marker density of 2700 SNPs per Mb. STRUCTURE and dendrogram showing genetic relationships revealed two recognizable groups with a very weak differentiation between them (*Fst* = 0.041) (Figure S3 and Table S1).

**Figure 5:**
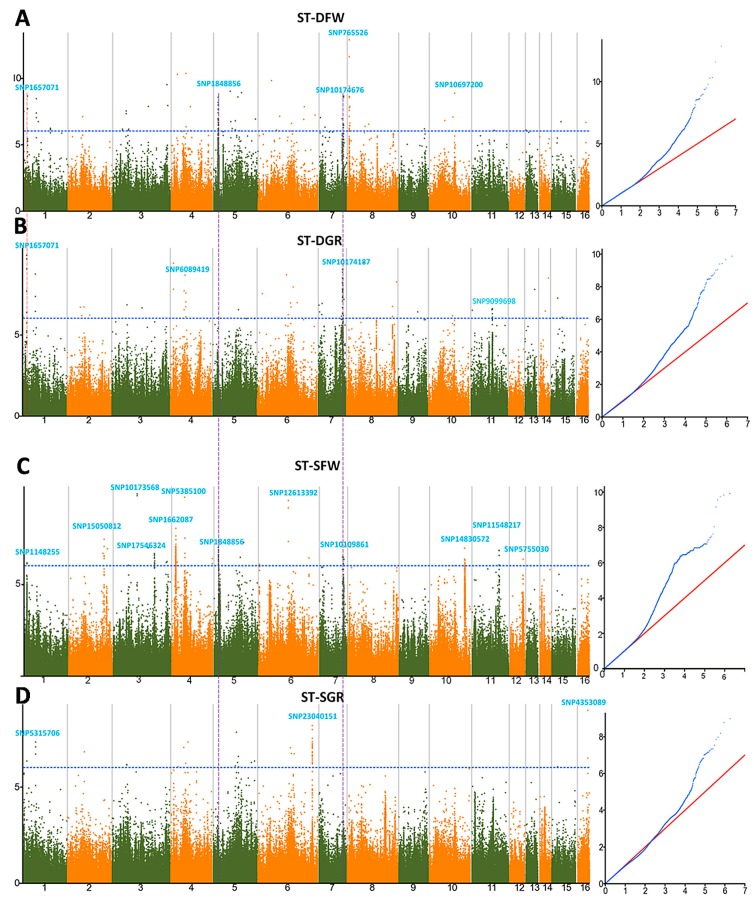
Manhattan plots and QQ plots of genome-wide association studies using the Mixed model for drought and salt tolerance indexes. (**A**) ST-DFW; (**B**) ST-DGR; (**C**) ST-SFW; (**D**) ST-SGR. The significant trait-associated QTLs commonly identified for the two indexes of the same stress are highlighted with dotted red lines and those commonly identified for drought and salt stresses are highlighted with dotted purple lines. QQ plot: Quantile-Quantile plots.

To get insight into the genetic variants associated with drought and salt tolerances at the germination stage in sesame, a GWAS was conducted for stress tolerance index on four traits (Figure 5). In regard to the PEG-induced drought stress, a total of 132 significant SNPs (−log_10_ (*p*) > 6) were uncovered, with 73 and 59 significant SNPs for ST-DFW and ST-DGR, respectively (Figure 5A,B). These SNPs were located on the linkage groups (LGs) 1, 4, 5, 7, 8, 10 and 11. The phenotypic variance explained (PVE) values of the peak SNPs were between 4.93% (SNP10174676) and 10.45% (SNP765526). Because of the dense coverage of SNPs in the whole genome, we assumed that the peak significant SNPs from a true association must be in a strong LD with the surrounding SNPs. Hence, this has led us to retain five QTLs (*qDFW1.1*, *qDFW5.1*, *qDFW7.1*, *qDFW8.1* and *qDFW10.1*) and four QTLs (*qDGR1.1*, *qDGR4.1*, *qDGR7.1* and *qDGR11.1*) for ST-DFW and ST-DGR, respectively. Between both indexes, we found two common QTLs regions (*qDFW1.1* similar to *qDGR1.1* and *qDFW7.1* similar to *qDGR7.1*) located on LG1 and LG7, led by the SNPs SNP1657071 and SNP10174187, respectively.

Concerning the NaCl-induced salt stress, we identified in total 120 significant SNPs (−log_10_ (*p*) > 6) including 90 and 30 significant SNPs for ST-SFW and ST-SGR, respectively (Figure 5C,D). Their PVE values ranged from 2.92% (SNP1848856) to 7.7% (SNP11548217). These SNPs were distributed on all the LGs except for the LGs9, 13 and 14. Following the same assumption described above, we identified 12 QTLs (*qSFW1.1*, *qSFW2.1*, *qSFW3.1*, *qSFW3.2*, *qSFW4.1*, *qSFW4.2*, *qSFW5.1*, *qSFW6.1*, *qSFW7.1*, *qSFW10.1*, *qSFW11.1* and *qSFW12.1*) for ST-SFW located on LGs 1, 2, 3, 4, 5, 6, 7, 10, 11 and 12 (Figure 5C). Similarly, three QTLs (*qSGR1.1*, *qSGR6.1* and *qSGR16.1*) were retained for ST-SGR located on LGs 1, 6 and 16 (Figure 5D). In contrast to drought stress indexes, no common QTL was found for the two salt tolerance indexes.

By comparing the results from drought and salt GWAS, we found only two common QTLs (*qSFW5.1* similar to *qDFW5.1* and *qSFW7.1* similar to *qDGR7.1*) associated with the stress indexes. These QTLs were linked to the SNPs SNP1848856 and SNP10174187 located on the LG5 and LG7, respectively.

### 3.4. Allelic Effects of the Associated SNPs on the Salt and Drought Tolerance Indexes in Sesame

**Figure 6:**
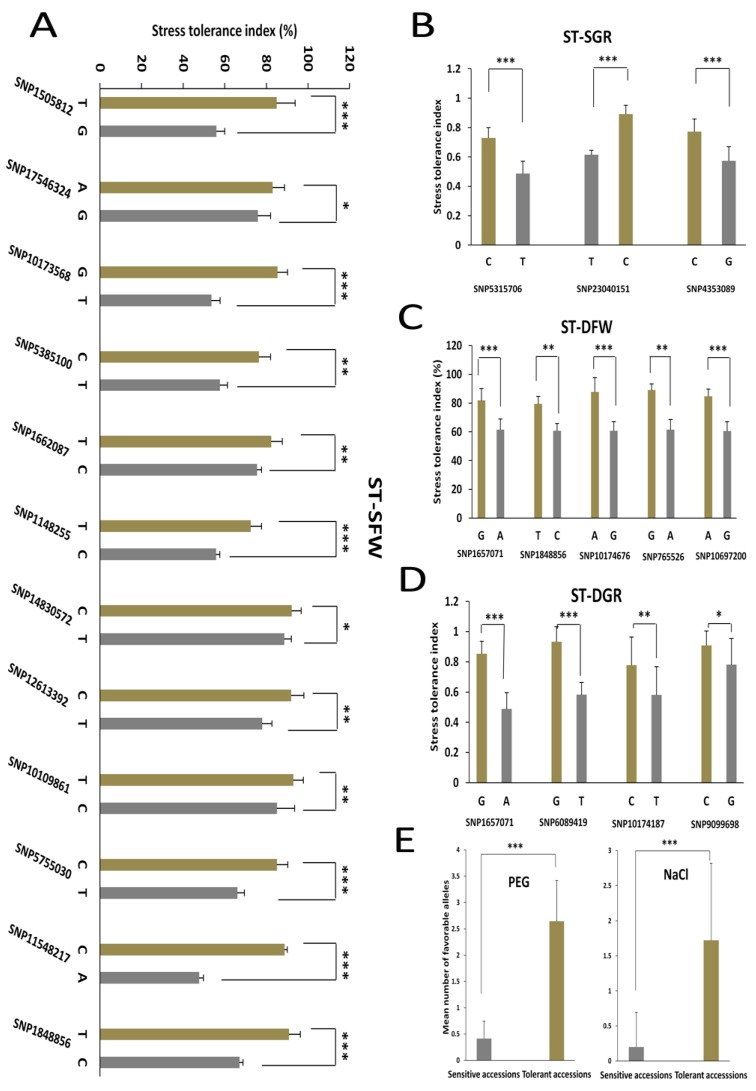
Allelic effects of the peak SNPs on NaCl-induced salt stress and PEG-induced drought tolerance indexes. (**A**) ST-SFW (%); (**B**) ST-SGR; (**C**) ST-DFW (%); (**D**) ST-DGR; (**E**) Mean number of favorable alleles identified in the tolerant and sensitive accessions to drought and salt stresses. *, **, *** significant difference at *p* < 0.05, *p* < 0.01, *p* < 0.001, respectively. For each SNP, the first allele is the reference allele and the second allele is the variant. Gray bars represent the favorable allele while the green bars represent the unfavorable allele. Error bars represent standard deviation.

The distributions of the stress tolerance indexes were further examined in individuals that carried each allele of the peak SNPs (Figure 6). We defined as favorable, the allele at the peak SNP which increases stress tolerance indexes. In general, the non-reference alleles at the peak SNPs were found to be the favorable alleles. Only the reference allele cytosine “C” at the SNP SNP23040151 significantly associated with ST-SGR, was identified as the favorable allele over the variant thymine “T” allele. In addition, there was no significant difference between the groups of accessions with the favorable alleles and the groups of accessions harboring the unfavorable alleles at the SNP SNP17546324, SNP14830572, SNP12613392, SNP4353089, SNP1848856 and SNP9099698 (Figure 6A–D).

The tolerant and sensitive groups of sesame accessions under PEG-induced drought stress (Table 2) were compared for variation and enrichment of favorable alleles at the peak SNPs associated with ST-DFW and ST-DGR. As shown in Figure 6E, the tolerant accessions harbored significantly more favorable alleles (ranging from one to seven, with a mean of three favorable alleles) than the sensitive group (ranging from zero to two, with a mean of zero favorable allele). The accession G440 harboring the highest number of favorable alleles (seven) has the best drought tolerance index (ST-GR and ST-FW).

Similarly, under NaCl-induced salt stress, there was a significant difference between the two groups (tolerant vs. sensitive). The tolerant accessions harbored more favorable alleles (ranging from one to five, with a mean of two favorable alleles) than the sensitive accessions (ranging from zero to two, with a mean of zero favorable allele). The accession G430 holds five favorable alleles and was one of the most salt tolerant genotype based on values of ST-SGR and ST-SFW.

### 3.5. Assigning Significant SNPs Associated with Drought and Salt Tolerance to Potential Candidate Genes

To assess the putative candidate genes associated with the significant SNPs for salt and drought tolerances in sesame, we retrieved all genes in the 88 kb window (LD region) around each peak SNP. A total of 241 and 151 genes were found for salt and drought stresses, respectively (Table 3, Table 4, Tables S3 and S4). The gene number around the peak SNPs ranged from seven to 33 for the drought tolerance index and from six to 32 for the salt stress index.

**Figure 7:**
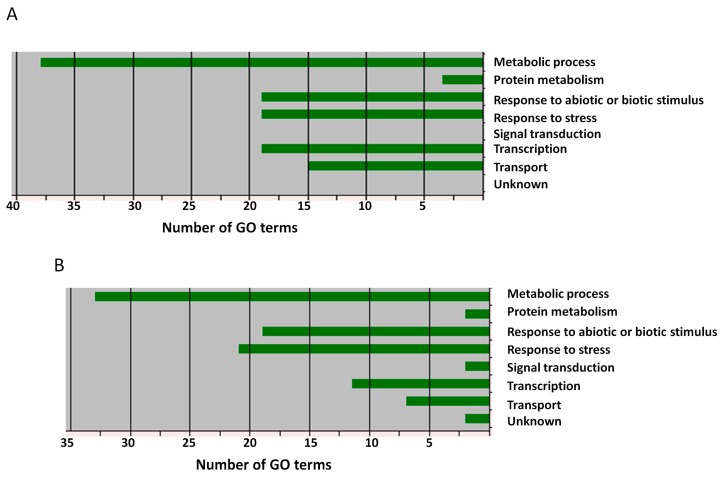
Biological process enrichment of the putative candidate genes. (**A**) PEG-induced drought stress; (**B**) NaCl-induced salt stress. GO: Gene Ontology.

Based on the annotation information of the retrieved genes and the functions described for their homologs in *A*. *thaliana*, we subsequently retained 13 putative candidate genes associated with PEG-induced drought tolerance (Table 3 and Table S5) and 27 potential candidate genes for NaCl-induced salt tolerance in sesame (Table 4 and Table S6). These genes encode transcription factors, antioxidative enzymes, osmoprotectants and are involved in hormonal biosynthesis or ion sequestration. Six common genes (*SIN_1007701*, *SIN_1007708*, *SIN_1007698*, *SIN_1008841*, *SIN_1008842* and *SIN_1009337*) were identified for drought and salt stresses. Gene ontology analysis of the potential candidate genes established that they are related to stress responses (Figure 7). Both drought and salt potential candidate genes were predominantly enriched in metabolic process, response to abiotic or biotic stresses and response to stress (Figure 7A,B).

## 4. Discussion

### 4.1. Drought and Salt Responses Are Governed by Different Genetic Components in Sesame

Drought and salinity stresses determine the primary cause of worldwide crop loss [56]. The germination stage is one of the crucial stages of the sesame crop growth cycle especially when it is confronted with abiotic stresses including waterlogging, drought and salt [9]. Although some QTLs and candidate genes were previously reported for waterlogging tolerance in sesame [57,58], very limited researches have been conducted concerning drought and salt stresses [2]. In this study, we observed that most of the sesame accessions were moderately tolerant to drought and salinity stresses as previously reported by several authors [5,6,14,19,21,23]. We noticed that the responses of sesame accessions to drought and salinity stresses were quite different based on stress tolerance indexes. This is further supported by the weak correlation between salt and drought tolerance indexes. Patade et al. [59,60] and Lokhande et al. [61] reported similar observations in sugarcane and *Sesuvium portulacastrum*, respectively. They demonstrated that PEG-induced osmotic stress and NaCl-induced salinity stress trigger differential biochemical and physiological responses. In fact, plants exposed to salt stress accumulate saline ions as an osmoticum stored in the vacuoles to prevent toxicity [62]. Additionally, they also synthesize compatible solutes such as proline, polyols, glycine betaine etc., for cellular osmotic adjustment [63]. In contrast, plants exposed to dehydration stress solely rely on the synthesis of compatible solutes for maintenance of the cell turgor (Lokhande et al. [61]).

Based on the phenotypic observations, we speculate that the genetic bases of drought and salt responses in sesame are different. Interestingly, the GWAS results corroborated well our hypothesis since, only two of the 20 identified QTLs were common to both abiotic stresses. In *Arabidopsis*, Kreps et al. [64] also showed that the transcriptomes involved in several abiotic stresses including cold, salt and drought were principally stimulus specific. Our results suggested that the genes controlling drought and salt stresses belong to fundamentally different genetic pathways in sesame.

### 4.2. GWAS Is an Effective Approach to Identify Functional SNPs and Candidate Genes for Drought and Salt Tolerances in Sesame

The modest LD decay rate in sesame and the high marker density coupled with the low genetic differentiation of our association panel are advantageous for GWAS implementation in this study. Most of the significant associations occurred in clusters of SNPs, delineating some important genomic regions governing drought and salt responses. In total, we identified 20 QTLs with modest effects for drought and salt tolerance indexes at the germination stage in sesame. The number of significant associated SNPs and their phenotypic contributions are similar to reports of salt and drought tolerances in *Brassica napus*, alfalfa and soybean [31,36,38]. The QTLs are distributed on 12 out of the 16 LGs of the sesame genome, suggesting that salt and drought responses are complex traits which implicate several genomic regions [56]. Moreover, these significant QTL regions harbored a very limited number of genes which is crucial to rapidly pinpoint the causative genes. Through classical bi-parental QTL mapping for plant height [65] and for yield related traits [66] in sesame, QTLs expanding on very large genomic regions and harboring hundreds of genes were discovered. Spotting the causative genes that govern these complex agronomic traits seems to be a daunting challenge. Therefore, GWAS is a more effective approach to dissect complex traits as compared to the classical bi-parental QTL mapping in sesame.

The favorable alleles at the peak SNPs improve tolerance under drought and salt treatments. We found more favorable alleles in the drought-tolerant accessions than in the salt-tolerant accessions, which may explain why sesame is naturally more tolerant to drought than salinity stress [16]. In addition, the accessions with the highest number of favorable alleles at the peak SNPs were the most tolerant. Knowing that marker-based allele pyramiding is very effective in crop improvement [67,68], we propose to confirm in future studies, whether the favorable alleles would have a positive pyramiding effect in improving drought and salt tolerances in sesame.

### 4.3. Discovering New Functional Genes for the Enhancement of Drought and Salt Tolerances in Sesame

To date, only few candidate genes are available for drought tolerance enhancement in sesame [10,11,12,69,70]. In contrast to drought, no gene or functional molecular marker is available for the improvement to salinity tolerance in sesame [2]. Here, we unraveled 13 and 27 potential candidate genes associated with the stress tolerance indexes for PEG-induced drought and NaCl-induced salinity, respectively. Most of the potential candidate genes are the homologs of well-described genes related to abiotic stress responses or plant development processes in the model species *Arabidopsis*. For example, the gene *SiOPR3* (*SIN_1024693*) detected for drought stress in sesame is the homolog of the gene *AT2G06050* (*ATOPR3*) in *Arabidopsis*. *ATOPR3*, an essential component of the jasmonic acid biosynthesis, is needed for the increase of abscisic acid in desiccating *Arabidopsis* roots which lead to a high drought tolerance [71]. Another interesting gene identified for drought is *SiWRKY69* (*SIN_1019661*). This gene is around the SNP SNP765526 with the highest PVE (10.45%). The homolog of *SIN_1019661* in *Arabidopsis* is *AT3G58710*, described as a non-memory gene functioning in response to dehydration stress. *AT3G58710* was constitutively and significantly up-regulated (>6-fold the expression level in the control plants) under multiple drought stresses [72]).

Between ST-DGR and ST-DFW, we detected a common QTL (*qDGR1.1* similar to *qDFW1.1*) located on the LG1. By examining the potential candidate genes in this genomic region, we retrieved the gene *SiCCD8* (*SIN_1021566*), the homolog of the *Arabidopsis* gene *AT4G32810* (*ATCCD8*). *ATCCD8* functions as a carotenoid cleavage dioxygenase and is involved in plant growth especially the hypocotyl development under several stress conditions [73]. The peak associated SNP SNP1657071 was located in an intron of *SiCCD8* with the allele ‘‘G’’ identified as the favorable allele. We infer that this intronic SNP may affect alternative splicing of the *SiCCD8* mRNA, its expression level and ultimately may alter the seed germination and the seedling growth potential under drought stress in sesame.

Concerning the salt stress, we also discovered several promising genes. In the QTL *qSFW2.1* led by the SNP SNP15050812, eight potential candidate genes which function in salt stress responses were uncovered. The gene *SiMLP31* (*SIN_1021337*) is annotated as a MLP-like protein which is the homolog of *Arabidopsis* gene *MLP31* (*AT1G70840*). According to Rajjou et al. [74], *MLP31* is implicated in salicylic acid synthesis which markedly improves seed germination and seedling vigor under salt stress. Based on these results, we speculate that the gene *SiMLP31* may hold similar attributes in sesame. Another interesting gene detected in this QTL region is *SiANTH* (*SIN_1021330*), the homolog of the gene *ANTH* (*AT2G01600*) in *Arabidopsis*. *ANTH* is a phosphatidic acid-binding protein which is recruited to membranes in response to salt stress in *Arabidopsis* roots [75]. Furthermore, a tandem array of several sesame Peroxidase encoding genes (*SiPOD*) was found in the same QTL region. These genes (*SIN_1021330*, *SIN_1021327*, *SIN_1021326*, *SIN_1021325*, *SIN_1021324*, *SIN_1021323* and *SIN_1021322*) may have resulted from a whole genome duplication event [76] and are likely to play the same function since all of them have the same homolog in *Arabidopsis* (*AT1G14550*). *AT1G14550* is reported to be involved in several abiotic stresses including drought, hypoxia and salt stress [77,78]. Plant peroxidases have been ascribed a variety of biological functions, including hydrogen peroxidase detoxification, lignin biosynthesis, hormonal signaling and stress response [79]. Under abiotic stresses such as salt, drought, cold, heat etc., plant accumulates excessive amount of reactive oxygen species (ROS) in cells which damages many cellular components. Peroxidase (POD) plays key roles in cellular ROS detoxification and is therefore a cardinal element of the plant antioxidant defense [80]. Based on these results, we deduce that this genomic region in the LG2 might be an important QTL for salt tolerance in sesame.

Identifying some QTLs associated with multiple desirable traits is highly sought in plant improvement strategies. Here, we found two QTLs (*qSFW5.1* similar to *qDFW5.1* and *qSFW7.1* similar to *qDGR7.1*) commonly associated with drought and salt tolerance indexes which may be useful for the development of sesame varieties tolerant to both stresses. In the first QTL region located on the LG5, we uncovered three promising candidate genes. Within them, the gene *SiHKT1* (*SIN_1007698*) is a sodium transporter crucial for plant survival under salt stress. Mounting evidence has highlighted this gene associated with ion content as an essential component in salinity tolerance in *Arabidopsis* and other plant species [81,82,83]. It is worth mentioning that by using GR and FW data under control condition for GWAS analysis, we were unable to detect the QTL region harboring *SiHKT1* (Data not shown). Therefore, identifying *SiHKT1* with the stress tolerance indexes data suggests that *SiHKT1* is not involved in sesame germination but in sesame response to salt stress at the germination stage. Nonetheless, whether this gene also functions in drought response in sesame or there is another gene in this QTL which is involved in drought response, is still unclear and will need additional analysis. In the second QTL located on LG7 common to both stresses, we also detected three potential candidate genes. Within these genes, *SiDREB2A* (*SIN_1009337*) that is the homolog of the gene *AtDREB2A* (*AT5G18450*) in *Arabidopsis* is well characterized for its involvement in drought and salt tolerances in several species [84,85,86,87].

To capture more effective candidate genes, we will conduct in future studies, RNA-seq analyses under PEG and NaCl stresses. Also, transgenic experiments and gene-based association analysis will help to shed more light on the functions of the candidate genes as well as the genetic variants altering their expression levels. Additionally, we will transform the significant peak SNPs into allele-specific markers such as Cleaved Amplified Polymorphic Sequences or Kompetitive allele specific PCR, in order to examine their potency as diagnostic molecular markers for sesame breeding programs.

## 5. Conclusions

In this study, stress tolerance indexes for four traits related to PEG-induced drought stress and NaCl-induced salt stress were assessed using a worldwide panel of sesame accessions at the germinating stage. Most of the accessions were moderately tolerant to both stresses. In addition, we found that sesame accessions respond differently to drought and salt stresses. GWAS revealed 20 QTLs including nine QTLs for drought and 15 QTLs for salt, with modest phenotypic contributions. Only two common QTLs were identified for both stresses, suggesting that the genes controlling these two abiotic stresses belong to fundamentally different genetic pathways. In total, 13 potential candidate genes associated with drought responses and 27 potential candidate genes for salt response were uncovered. These genes encode transcription factors, antioxidative enzymes, osmoprotectants and are involved in hormonal biosynthesis or ion sequestration. Interestingly, most the potential candidate genes are described to be involved in responses to abiotic stresses. Altogether, the phenotyped biomaterials, the discovered functional SNPs and the potential candidate genes for drought and salt responses will be helpful for marker-assisted breeding programs aimed at enhancing salt and drought tolerances in sesame cultivars.

## Figures and Tables

**Table 1:** Phenotypic variation of GR and FW under control and stress conditions.

Trait	Treatment (T)	Mean	SD	Range	Variance	Skewness	Kurtosis	A	T	A × T
**GR**	Distilled water	40	9.40	33–50	88.40	−1.75	3.65	***	***	***
	PEG stress	25.24	4.60	0–50	112.42	−1.46	1.94			
	Salt stress	24.23	3.46	0–50	109.51	−1.41	1.66			
**FW (g)**	Distilled water	1.49	0.52	1.08–2.93	0.26	−0.28	−0.30	***	***	***
	PEG stress	0.66	0.18	0.0.5–1.61	0.08	−0.27	0.00			
	Salt stress	0.7	0.16	0.05–1.92	0.22	0.23	0.05			

A = Accessions, T = Treatments, GR = number of germinated seeds and FW = Fresh weight of the seedlings. *** Significant difference at *p* < 0.001.

**Table 2:** Distribution of the accessions according to their tolerance levels to salt and drought stresses.

Stress	Tolerance Levels	Number of Accessions	Percentage (%)	Mean ST-GR	Mean ST-FW
	Sensitive	195	40	0.76	66.28
Salt	Moderate	230	47	0.87	81.83
	Tolerant	65	13	1.17	100.62
	Sensitive	176	36	0.78	50.31
PEG	Moderate	207	42	0.94	56.75
	Tolerant	107	22	1.06	91.14

**Table 3:** SNPs significantly associated with PEG-induced drought tolerance indexes at the germination stage in sesame.

Trait	LG	QTL	Peak SNP	–log10 (*p*)	Ref Base	SNP Base	MAF	Genes in LD	PVE (%)	Candidate Gene ID	Gene Name
**ST-DFW**	LG1	*qDFW1.1*	SNP1657071	8.63	A	G	0.06	24	7.23	*SIN_1021558*	*SiCCD8*
										*SIN_1021566*	*SiEMF1*
	LG5	*qDFW5.1*	SNP1848856	8.55	C	T	0.07	24	6.89	*SIN_1007701*	*SiGPAT3*
										*SIN_1007708*	*SiAGL37*
										*SIN_1007698*	*SiHKT1*
	LG7	*qDFW7.1*	SNP10174676	6.54	G	A	0.04	27	4.93	*SIN_1008841*	*SiGDH2*
										*SIN_1008842*	*SiCYP76C7*
										*SIN_1009337*	*SiDREB*
	LG8	*qDFW8.1*	SNP765526	12.88	A	G	0.03	33	10.45	*SIN_1019660*	*SiRABA1D*
										*SIN_1019661*	*SiWRKY69*
	LG10	*qDFW10.1*	SNP10697200	8.85	G	A	0.06	7	7.67	NA	NA
**ST-DGR**	LG1	*qDGR1.1*	SNP1657071	8.63	A	G	0.06	24	7.23	*SIN_1021566*	*SiEMF1*
										*SIN_1021558*	*SiCCD8*
	LG4	*qDGR4.1*	SNP6089419	8.66	T	G	0.07	10	7.22	*SIN_1001572*	*SiGRV2*
	LG7	*qDGR7.1*	SNP10174187	9.01	T	C	0.03	26	6.61	*SIN_1008842*	*SiCYP76C7*
										*SIN_1009337*	*SiDREB*
										*SIN_1008841*	*SiGDH2*
	LG11	*qDGR11.1*	SNP9099698	6.59	G	C	0.06	25	5.3	*SIN_1024695*	*SiGRF5*
										*SIN_1024693*	*SiOPR3*

PVE (%): Phenotypic variance explained, refbase: reference allele, SNPbase: non-reference allele, MAF: Minor Allele Frequency. LG: Linkage Group.

**Table 4:** SNPs significantly associated with NaCl-induced salt tolerance indexes at the germination stage in sesame.

Trait	LG	QTL	Peak SNP	–log10 (*p*)	REF BASE	SNP Base	MAF	Genes in LD	PVE (%)	Candidate Gene ID	Gene Name
**ST-SFW**	LG1	*qSFW1.1*	SNP1148255	6.16	C	T	0.05	20	3.93	*SIN_1021624*	*SiLHCB6*
	LG2	*qSFW2.1*	SNP15050812	7.45	G	T	0.05	21	8.03	*SIN_1021337*	*SiMLP31*
										*SIN_1021330*	*SiANTH*
										*SIN_1021327*	*SiPOD*
										*SIN_1021326*	*SiPOD*
										*SIN_1021325*	*SiPOD*
										*SIN_1021324*	*SiPOD*
										*SIN_1021323*	*SiPOD*
										*SIN_1021322*	*SiPOD*
	LG3	*qSFW3.1*	SNP17546324	6.66	G	A	0.03	16	4.04	*SIN_1015378*	*SiHSFA1*
	LG3	*qSFW3.2*	SNP10173568	9.92	T	G	0.08	15	8.32	*SIN_1017475*	*SiDUF538*
	LG4	*qSFW4.1*	SNP5385100	9.74	T	C	0.05	6	7.45	*SIN_1018894*	*SiCC-NBS-LRR*
	LG4	*qSFW4.2*	SNP1662087	8.04	C	T	0.06	8	6.52	*SIN_1008463*	*SiUDG*
	LG5	*qSFW5.1*	SNP1848856	8.55	C	T	0.07	24	2.92	*SIN_1007701*	*SiGPAT3*
										*SIN_1007708*	*SiAGL37*
										*SIN_1007698*	*SiHKT1*
	LG6	qSFW6.1	SNP12613392	9.5	T	C	0.06	6	6.52	*SIN_1018616*	*SiNAC43*
	LG7	qSFW7.1	SNP10109861	6.45	C	T	0.04	9	5.88	*SIN_1008841*	*SiGDH2*
										*SIN_1008842*	*SiCYP76C7*
										*SIN_1009337*	*SiDREB*
	LG10	qSFW10.1	SNP14830572	6.95	T	C	0.03	23	4.91	*SIN_1026087*	*SiCP24*
	LG11	qSFW11.1	SNP11548217	6.83	A	C	0.04	32	7.7	*SIN_1013032*	*NA*
	LG12	qSFW12.1	SNP5755030	6.37	T	C	0.06	16	6.68	*SIN_1006749*	*SiWRKY14*
										*SIN_1006753*	*SiLSD1*
**ST-SGR**	LG1	*qSGR1.1*	SNP5315706	7.33	T	C	0.06	10	5.91	*SIN_1026318*	*SiXXT5*
	LG6	*qSGR6.1*	SNP23040151	8.18	C	T	0.03	24	8.45	*SIN_1022410*	*SiXTH15*
	LG16	*qSGR16.1*	SNP4353089	8.98	G	C	0.07	10	7.45	*SIN_1003799*	*SiG6PD1*

## Supplementary Materials

The following are available online at http://www.mdpi.com/2073-4425/9/2/87/s1, Figure S1: Responses of tolerant and sensitive sesame genotypes to PEG 6000 at the concentration 15%; Figure S2: Responses of tolerant and sensitive sesame genotypes to NaCl at the concentration 60 mM; Figure S3: Population genetics of the association panel. (A) Neighbor-joining tree of all the varieties calculated from whole-genome SNPs revealed two recognizable groups colored in red and purple; (B,C) Estimated population structure in the association assessed by STRUCTURE. Each individual is represented by a thin vertical bar, partitioned into k = 2 colored segments representing two subpopulations; Table S1: Full list of the 490 sesame accessions used in this study, their origins, breeding status, sequencing information, dendrogram grouping, salt and drought tolerance status; Table S2: List of the 15 accessions used for preliminary salt and drought screening experiments; Table S3: Full list of the genes detected around the peak SNPs for PEG-induced drought stress in the sesame association panel; Table S4: Full list of the genes detected around the peak SNPs for NaCl-induced salt stress in the sesame association panel; Table S5: List of the significant peak SNPs and annotation of associated potential candidate genes for PEG-induced drought stress; Table S6: List of the significant peak SNPs and annotation of associated potential candidate genes for NaCl-induced salt stress.
